# The Communicable Fevers and Their Treatment

**Published:** 1879-09

**Authors:** 


### THE COMMUNICABLE FEVERS AND THEIR TREATMENT.

In a lecture recently delivered at Belevue Hospital by Dr. W. H. Thompson, and reported in *The Boston Medical and Surgical Journal*, the following points referable to communicable fevers are presented :

In others of these diseases beside diphtheria there are special indications. Thus in scarlatina and typhoid we are always to be on our guard against the occurrence of otitis. Just as soon as the patient complains of any pain in the ear, the best thing is to inject into it oil of peppermint, which, like that of wintergreen, is a disinfectant. In typhoid there are two classes of cases in which the ear is usually affected. In the one, inflammation proceeds up from the throat into the internal ear, while in the other the ear trouble is simply a part of the desquamative process. In a case of typhoid I dislike very much to find diphtheria as a complication, since it is extremely likely to result in permanent deafness. I think I have warded this off in quite a number of instances, however, by the method of free irrigation of the throat, previously alluded to. In scarlatina, if the ear is affected early, we have external otitis, and external syringing will be found useful ; but if the difficulty comes on later, it is the result of the spreading of the inflammatory process from the throat, and we have otitis media. Here it is better to use the method of irrigating the throat ; in severe cases of scarlatina, even where there is no otitis or diphtheria, I would strongly recommend its employment.

But we have other difficulties in scarlatina beside otitis, and prominent among them are delirium and convulsions. These you will find to be due to the high temperature present. Therefore, whenever I meet with any great increase in the body heat, I am in the habit of combating it immediately with cold-water applications. In scarlatina a high temperature is more dangerous than in any other affection with which I am acquainted. One hundred and six degrees almost always prove fatal, one hundred and five are very bad, and even one hundred and four are dangerous. In children the cold-pack is the best means of refrigeration, and when delirium or convulsions occur, cold is especially to be applied to the head.

From the very beginning of an attack of scarlatina we should never neglect to use inunction of the whole body three times a day. This answers quite a number of purposes, and is particularly useful in relieving itching and restlessness, and consequently enables us to keep the patient much more quiet than we could otherwise do. The itching is always annoying, because in this disease there is such a general inflammation of the skin, and hence the application of the oil is very grateful. If it is quite severe, lime-water liniment had better be employed, as the alkali renders the inunction more soothing. Another end that this procedure fulfills is the warding off, to a considerable extent, of renal complication ; and it is often of still further service in preventing glandular trouble, as well as those prolonged affections of the mucous membranes, such as conjunctivitis and ozena, which are often so miserable a feature of scarlatina. Glandular implication, indeed, is always secondary to difficulty about the throat, ear, or nose, and hence the best way to avoid it is to use faithful inunction in combination with throat irrigation.

Lastly, as to scarlatina nephritis. In every case a prodigeous amount of uric acid is given off by the kidneys. In the urine of a patient suffering from the disease, I have sometimes seen the test-tube one-third filled with a cloudy deposit looking like mucus, but in reality made up of the crystals of uric acid. For some time past I have been in the habit of using the wine of colchicum in cases of scarlet fever accompanied by kidney complication, and I have found the treatment, which I believe is original with myself, of material benefit. The dose generally employed is about five drops.

A few words now in regard to typhoid fever. This is the longest of the fevers, and, being characterized by the most continued pyrexia, it differs from the others in the immense amount of heat ordinarily given off during its course. The high temperature is long-continued and steady, while in the others it is shorter or intermittent (as in malarial fever), and hence in it there is, as a rule, more burning up of the materials of the body. There being thus a greater waste of the vital forces, it is the most exhausting of all the fevers. Consequently, in typhoid one of the leading indications is to employ apyretics, and I am a strong advocate of the use of cold as well as quinine and other agents capable of reducing temperature in its treatment.

Along with such measures, proper alimentation must be kept up steadily from the beginning to the end of the attack ; for there is great danger of the patient's giving out from pure starvation. The food must always be administered systematically, and it is absurd to commence your treatment with stimulus, which should always be reserved until it is really needed. To do good, it should be given only when the critical period arrives. Stimulation is for a special time, and you do not order it to-day for an operation that is to be performed next week, for instance. It is imperatively necessary, however, that you should commence the feeding at once. The stomach is so weak that it is like that of an infant, and hence the only appropriate diet is milk, which it is better to dilute one half with lime-water. About a drachm of pepsin a day is also advisable, to assist its digestion, and bismuth is of service in preventing putrefactive changes in the intestines by its local action. It may, perhaps, be well to commence the treatment with a cathartic, but, beside the simple remedies just mentioned, I see no need for any further medication.

In typhus fever there is a greater tendency to cerebral trouble than in typhoid. Ice or cold applications should therefore be early placed upon the head, and should be kept up as long as any headache persists. General inunction is more necessary in this disease than in typhoid, and is, indeed, scarcely less so than in scarlatina itself.

